# The effects of workplace stressors on muscle activity in the neck-shoulder and forearm muscles during computer work: a systematic review and meta-analysis

**DOI:** 10.1007/s00421-013-2602-2

**Published:** 2013-03-05

**Authors:** B. H. W. Eijckelhof, M. A. Huysmans, J. L. Bruno Garza, B. M. Blatter, J. H. van Dieën, J. T. Dennerlein, A. J. van der Beek

**Affiliations:** 1 Department of Public and Occupational Health and the EMGO Institute for Health and Care Research, VU University Medical Center, Van der Boechorststraat 7, 1081 BT Amsterdam, The Netherlands; 2 Body@Work Research Center on Physical Activity, Work and Health, TNO-VU/VUmc, Amsterdam, The Netherlands; 3 Department of Environmental Health, Harvard University, 677 Huntington Avenue, Boston, MA 02115 USA; 4 Netherlands Organisation for Applied Scientific Research, TNO, Polaris Avenue 151, 2130 AS Hoofddorp, The Netherlands; 5 MOVE Research Institute Amsterdam, Faculty of Human Movement Sciences, VU University Amsterdam, Van der Boechorststraat 7, 1081 BT Amsterdam, The Netherlands; 6 Department of Physical Therapy, Bouvé College of Health Sciences, Northeastern University, 360 Huntington Avenue, Boston, MA 02115 USA

**Keywords:** Psychosocial stress Work pace, Precision, Electromyography, Trapezius, Mouse use

## Abstract

Workplace stressors have been indicated to play a role in the development of neck and upper extremity pain possibly through an increase of sustained (low-level) muscle activity. The aim of this review was to study the effects of workplace stressors on muscle activity in the neck-shoulder and forearm muscles. An additional aim was to find out whether the muscles of the neck-shoulder and the forearm are affected differently by different types of workplace stressors. A systematic literature search was conducted on studies investigating the relation between simulated or realistic workplace stressors and neck-shoulder and forearm muscle activity. For studies meeting the inclusion criteria, a risk of bias assessment was performed and data were extracted for synthesis. Results were pooled when possible and otherwise described. Twenty-eight articles met the inclusion criteria, reporting data of 25 different studies. Except for one field study, all included studies were laboratory studies. Data of 19 articles could be included in the meta-analysis and revealed a statistically significant, medium increase in neck-shoulder and forearm muscle activity as a result of workplace stressors. In subgroup analyses, we found an equal effect of different stressor types (i.e. cognitive/emotional stress, work pace, and precision) on muscle activity in both body regions. In conclusion, simulated workplace stressors result in an increase in neck-shoulder and forearm muscle activity. No indications were found that different types of stressors affect these body regions differently. These conclusions are fully based on laboratory studies, since field studies on this topic are currently lacking.

**Electronic supplementary material:**

The online version of this article (doi:10.1007/s00421-013-2602-2) contains supplementary material, which is available to authorized users.

## Introduction

### Background

Computer work has become a key element in daily work for many people and is still growing. Neck and upper extremity pain is a common health problem among computer workers with prevalence rates of 25 % for the neck-shoulder and 15 % for the forearm region in Europe (de Kraker and Blatter 2005). Neck and upper extremity pain not only has serious consequences for the individual involved but also is associated with high costs for societies and employers, due to productivity loss and medical consumption. In the Netherlands, total yearly costs are estimated at 2.1 billion euro (Blatter et al. 2005).

Neck and upper extremity pain has a multifactorial origin. One category of risk factors for neck and upper extremity pain is workplace stress (Bongers et al. 2002; 2006), which is common in computer work. Workplace stressors include a wide variety of stressors, such as high work demands, high mental processing, high memory demands, performing multiple tasks at the same time, time pressure, low decision authority, low reward, and high efforts (e.g. Karasek et al. 1998; Siegrist et al. 1997). The etiological mechanisms of workplace stressors are not yet understood, but one of the mechanisms proposed is that these stressors increase sustained (low-level) muscle activity (Bongers et al. 2006) which in turn may lead to injury via overexertion. Two pathways may play a role. Firstly, muscle activity may be affected indirectly through a changed work style due to these stressors (e.g. increased work pace, high forces on the keyboard and mouse, and more awkward and sustained postures) (Harrington and Feuerstein 2010). Secondly, muscle activity may be affected directly without any change in posture or movement (Bloemsaat et al. 2005; Waersted 2000; Waersted and Westgaard 1996) due to increased arousal or due to more specific psychogenic mechanisms.

Indications have been found that the underlying mechanisms for developing neck-shoulder or forearm symptoms may be different (IJmker et al. 2007), related to the different pathways described above. Bloemsaat et al. (2005) found that an increased work pace resulted in higher muscle activity in the distal hand-arm region, whereas increased mental demands resulted in higher muscle activity in the proximal arm-shoulder region. Workplace stressors such as time pressure are likely to interfere with the task performance by increasing work pace, which in turn is likely to enhance the activity of the muscles controlling the operating hand. Stressors without this task-interfering component, such as high mental processing, may be more likely to enhance muscle activity of the posture controlling muscles of the neck and shoulders.

Many experimental laboratory studies have been conducted on this topic. Several found that a higher level of the simulated or realistic workplace stressors resulted in a higher muscle activity (e.g. Laursen et al. 2002; Mclean and Urquhart 2002; Wahlstrom et al. 2002). However, other studies did not find this relationship (e.g. Blangsted et al. 2004; Sandfeld and Jensen 2005), possibly due to a lack of statistical power. Other reasons for conflicting results between studies may have arisen because different types of stressors were used, some interfering with the task while others did not, or because different body regions were studied. Therefore, studying the effects of different types of workplace stressors separately may provide a better understanding of whether these stressors lead to increased muscle activity in the neck-shoulder muscles and/or the forearm muscles. To our knowledge, no systematic review on this topic has been published so far. The aim of our review is to answer the following two questions:

- Do workplace stressors increase muscle activity during simulated or realistic computer work?
- Do different types of workplace stressors affect neck-shoulder and forearm muscle activity differently during simulated or realistic computer work?

## Methods

### Literature search

To identify relevant articles providing information about the effects of workplace stressors on neck and upper extremity muscle activity, we performed systematic searches in the bibliographic databases PubMed, EMBASE.com, PsycINFO and The Cochrane Library from inception to April 04, 2011. A search strategy was developed for PubMed (see Online Resource 1) and adapted for other databases. Search terms included controlled terms from MeSH in PubMed, EMtree in EMBASE.com and Thesaurus terms in PsycINFO as well as free text terms. In The Cochrane library, we only used free text terms. Different blocks of search terms comprising ‘computer use’, ‘neck and upper extremity’, ‘biomechanics’, and ‘psychosocial stress’ were composed and used in AND combination (Online Resource 1). In addition, the references of the included articles, based on the selection criteria below, were checked for relevant titles that were not indicated by the electronic search.

### Selection of studies

The focus of the final literature review is smaller than that of the initial systematic search of articles. Given the large number of articles identified and the large variability between outcome measures in these studies, we decided to narrow down our research question to the effect on muscle activity alone. Since muscle activity can be influenced by the presence of pain (e.g. Westgaard 1999), we decided to only include data of healthy subjects. To obtain results that can be generalized to computer work, only studies involving computer work or a realistic simulation of computer work were included.

Articles were eligible for inclusion if they met the following criteria:

- Peer-reviewed full-text paper written in English, Dutch, German or French.
- Reported on muscle activity in the neck–shoulder or forearm muscles, measured by EMG, during a computer task with versus without induced workplace stressors.
  - A computer mouse, keyboard or simulated keys were used as input device.
  - The stressful condition was either induced by an experimenter or it covered realistic (occupational) stressors, indicated by self-report.
  - As outcome measure for muscle activity, mean or median EMG amplitudes were reported.
- Participants were free of pain and musculoskeletal symptoms in the neck and upper extremities or data were separately reported for symptom free participants.

To identify relevant articles, two reviewers (LE and JB) independently screened the studies resulting from the literature search. In a first selection round, screening was based on title only. Only when a title indicated that it was absolutely certain that the study concerned a topic different from the interest of this review, the study was excluded. In case the reviewers disagreed or were in doubt, the article remained included. Secondly, selection on title and abstract was carried out, while in a third selection round all remaining full-text articles were checked for eligibility. After every selection round the results of both reviewers were compared and disagreements were discussed. If disagreement remained, a third reviewer (MH) was consulted to reach consensus.

### Risk of bias assessment

To assess whether the results of some studies were more likely to be affected by bias than others, two reviewers (LE and JB) independently performed a risk of bias assessment on the included studies. Before the actual assessment was carried out, a pilot assessment was performed on a closely related (but not included) article and disagreement was discussed.

A checklist with assessment criteria relevant for (small) experimental trials was composed, including both internal validity aspects such as randomization, baseline muscle activity measurements, reliability, and confounding, and external validity aspects concerning generalizability (Table 1). Internal validity of the measurements, items 1 and 2, was based on the description of selection bias in the Cochrane handbook (Higgins and Green 2011). Internal validity of the intervention, items 3a, 3b, and 4, was based on the description of performance bias in the Cochrane handbook (Higgins and Green 2011). The items 5a–c concerning external validity were derived from Mazaheri et al. (2012). Item 4 evaluated whether an extra motor component was unintentionally introduced by the experimental manipulation that could have influenced the EMG outcomes, such as for example verbally responding during a memory task. Please note that this extra motor component does not refer to motor changes as part of the experimental manipulations, such as increased work pace or precision. Items scored either a full point (1, no potential bias), half a point (1/2, some potential bias), or no point (0, potential bias). If insufficient information on a particular item was provided in the paper, then it was scored with a question mark (?, don’t know). If an item was not relevant, then it was scored as not applicable (NA).

**Table 1:** Risk of bias checklist

		Scoring
	*Internal validity (measurements)*	
1	Was muscle activity before the control and experimental condition comparable (i.e. an equal baseline)?	A full point (1) was assigned if muscle activity was equal at baseline
2	Were the control and experimental conditions randomized?	A full point (1) was assigned if the control and experimental conditions were randomly assigned to the participants or balanced across participants
	*Internal validity (intervention)*	
3a	Did the experimental manipulation result in the intended increased stress level?	A full point (1) was assigned if the effect of the stress intervention was tested in the study and showed an increase Half a point (1/2) was assigned if a reference to a study that did was provided Half a point (1/2) was assigned if more than one intervention was performed and only in one of the two the effect of the stress intervention was tested in the study and showed an increase
3b	Do the stressful manipulations offer escape possibilities?	A full point (1) was assigned if it was *not* possible to ignore the stressful manipulation, and compensate by e.g. a decreased work pace Half a point (1/2) was assigned if more than one intervention was performed and only in one of the two it was not possible to ignore the stressful manipulation, and compensate by e.g. a decreased work pace
4	Did the experimental manipulation introduce an unintended extra motor component, influencing EMG outcomes?	A full point (1) was assigned if the experimental manipulation did *not* introduce an unintended extra motor component that influences the EMG outcomes
	*External validity*	
5a	Is the study population representative for computer workers?	A full point (1) was assigned if the study population was completely relevant for computer workers Half a point (1/2) was assigned if the study population was representative for subgroup of computer workers (e.g. only students or only men/women)
5b	Are the studied tasks representative for computer work?	A full point (1) was assigned if the studied task reflected realistic computer work
5c	Is the stress intervention representative for stressors in a realistic occupational setting?	A full point (1) was assigned if the intervention reflected realistic stressors

The results of both reviewers on all items were compared to identify disagreements. During a consensus meeting disagreements were solved. Finally, sub-scores for internal and external validity (% of maximum number of points scored per category), and a total score (% of maximum number of points scored of all items) were calculated per study as an indication of differences in the risk of bias across studies.

### Data extraction

From the included articles, information about the following aspects was extracted and summarized in a data extraction table (Online Resource 2): study population, study design, computer task, induced stressor(s), wash-out period, and muscles measured. Besides the data reported in the data extraction table, EMG data were extracted from the articles in order to perform a meta-analysis. From presented tables and figures, the mean or median EMG levels of muscles active during task performance were extracted. Muscles of interest in the neck-shoulder region were the upper trapezius muscle and neck extensors. Muscles of interest in the forearms were the wrist and finger extensors and flexors.

### Data synthesis

#### Meta-analysis

To evaluate the effect of workplace stressors on neck-shoulder and forearm muscle activity, all available EMG data from the included studies were synthesized in a meta-analysis. If multiple experimental conditions or muscle groups were reported in a single article, then data for each condition or muscle group were first combined into a single mean before running the overall analysis.

#### Effect size

Since EMG outcomes were expressed in different units, a standardized mean difference was used as effect size. In most cases, EMG was expressed as a percentage of the maximal voluntary contraction (%MVC). In one case, EMG was expressed as the percentage of a reference contraction in which a 1 kg dumb-bell was lifted for 2 s (Wahlstrom et al. 2002), in one case, EMG was expressed in micro volts (uV) (Kristiansen et al. 2009), and in one case, the expression of EMG was unclear (Rietveld et al. 2007).

Because sample size was expected to be small in many studies, Hedges’ *g* was chosen as the best effect size, since it corrects for possible slight overestimations in small samples (Borenstein et al. 2009). The effect size was defined as small if Hedges’ *g* < 0.33, medium if 0.33 ≤ Hedges’ *g* ≤ 0.55, and large if Hedges’ *g* > 0.55, based on Cohen’s *d* interpretations (Lipsey 1990). Mean effect sizes were calculated using meta-analysis software (Comprehensive Meta-Analysis, Biostat, 14 North Dean Street, Englewood, NJ 07631, USA). In the meta-analysis, we used a random-effects model within (sub)groups. Increased muscle activity in the stress condition compared to the control condition was set as a positive effect size.

In addition, we expected to include many studies with a cross-over design. Then a pre-post correlation coefficient is needed for calculating the effect size; Hedges’ *g*. A pre-post correlation coefficient of 0.65 was chosen, based on Mathiassen et al. (2002). They found a correlation coefficient of 0.60 for comparable EMG measurements, performed on different days. When measurements are performed on the same day, a higher correlation coefficient is expected, because electrodes are mounted only once. Almost all included articles in this review reported data of repeated measures collected on the same day.

#### Subgroup analyses

**Fig. 1:**
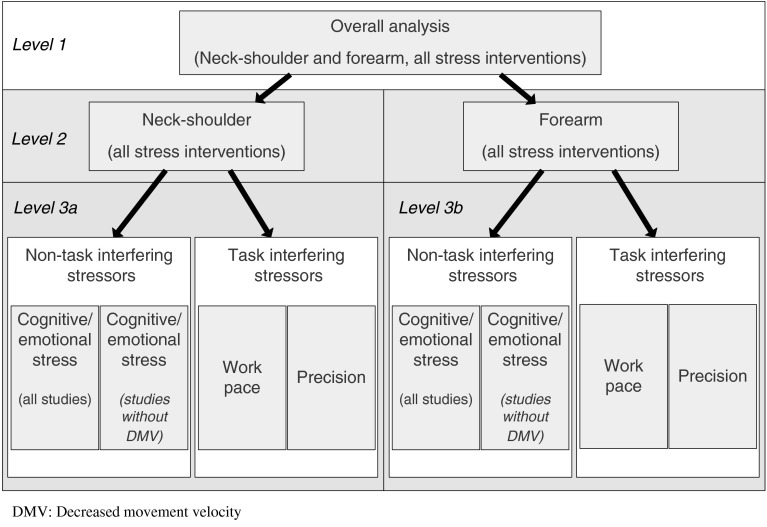
Overview of the different subgroup analyses that were conducted in the meta-analyses

To examine whether different types of workplace stressors affect neck–shoulder and forearm muscle activity differently (our second research question), subgroup analyses were performed with a minimum of three studies within a subgroup. These subgroup analyses are depicted schematically below (Fig. 1).

In the first level, analyses were stratified into two subgroups based on muscle location: neck–shoulder or forearm. Then, a second level of stratification within these two subgroups (neck–shoulder and forearm) was applied, based on the type of stressor in the experimental condition. The stress interventions were categorized into interventions that: (a) increase cognitive loading and/or emotional stress, (b) increase work pace, and (c) increase precision demands. The first category involves stressor types that do not interfere with the computer task directly and the latter two categories involve stressor types that do. Examples of stressors assigned to the category cognitive loading/emotional stress are the STROOP color word test, memory tests, mental arithmetic tests, complex reaction time tasks, mental pressure on good performance, lack of support, and simulated office noise/environmental distraction. Examples of stressors in the second category, i.e. increased work pace, are increased typing speed, increased key tapping rate, increased mouse clicking speed, and the instruction to work as quickly as possible. In the third category, i.e. increased precision demands, stressors such as high clicking accuracy (provoked by a reduced target size) and increased mouse gain are included. In some studies, the experimental condition included a combination of stressors from different categories. Studies in which the experimental condition involved a combination of subgroups were not included at this level of the subgroup analyses.

In order to answer our second research question properly, we decided to perform a cognitive/emotional stressor subgroup analysis, including only studies in which movement velocity did not decrease in the experimental condition. Decreasing movement velocity can be seen as an adaptation strategy to cope with the induced stressor. However, since it influences muscle activity (Birch et al. 2000), it is also a confounding variable for our research question. In the data extraction table (Online Resource 2), it is reported whether this adaptation strategy occurred in a study, and whether the study was included in this subgroup analysis.

Furthermore, a sensitivity analysis based on results of the risk of bias assessment (Tables 1, 2) was performed to evaluate whether studies with a high risk of bias compared to studies with a low risk of bias showed a different effect of the induced stressor on muscle activity.

**Table 2:** Results risk of bias assessment, in which 1 is a full point, 1/2 is half a point, 0 is no point, and NA is not applicable shoulder and forearm muscles separately

Authors	Internal validity (measurements)		Internal validity (intervention)			Sub-score (%)^a^	External validity			Sub-score (%)^a^	Total score (%)^b^
	1^c^	2^c^	3a^c^	3b^c^	4^c^		5a^c^	5b^c^	5c^c^		
Blangsted et al. (2003)**	?	NA	NA	1	1	67	1/2	1	1	83	75
Leyman et al. (2004)	?	1	1	0	1	60	1	1	1	100	75
Rietveld et al. (2007)	?	0	1	1	1	60	1	1	1	100	75
Ekberg et al. (1995) (main)	?	1	1	0	1	60	1/2	1	1	83	69
Gerard et al. (2002)	?	1	1	0	1	60	1/2	1	1	83	69
Hughes et al. (2007)	?	1	1	0	1	60	1/2	1	1	83	69
Szeto et al. (2005)**	?	0	1	1	1	60	1/2	1	1	83	69
Wahlstrom et al. (2002)	?	0	1	1	1	60	1/2	1	1	83	69
Wang et al. (2011)	?	0	1	1	1	60	1/2	1	1	83	69
Alkjaer et al. (2005)	?	1	1	0	1	60	1/2	1	1/2	67	63
Blangsted et al. (2004)	?	0	1/2	1	1	50	1/2	1	1	83	63
Ekberg et al. (1995) (pilot)	?	1	1	0	1	60	1/2	1	1/2	67	63
Visser et al. (2004)	?	1	1/2	1/2	1	60	1	0	1	67	63
Kristiansen et al. (2009)	?	1	0	1	1	60	1/2	0	1	50	56
Laursen et al. (2002)	?	1	1/2	1	1	70	1/2	1/2	0	33	56
McLean and Urquhart (2002)	?	0	1	0	1	40	1/2	1	1	83	56
Schnoz et al. (2000)	?	0	1	1	1	60	1/2	0	1	50	56
Szeto and Lin (2011)**	?	1	1/2	1/2	1	60	1/2	0	1	50	56
Waersted et al. (1994)***	?	1	1	0	1	60	1/2	0	1	50	56
Westad et al. (2004); Westgaard et al. (2006)*	?	0	1	0	1	40	1	1/2	1	83	56
Bloemsaat et al. (2005)	?	1	1/2	0	1	50	1/2	0	1	50	50
Finsen et al. (2001a, b)	?	0	1	0	1	40	1/2	1/2	1	67	50
Johnston et al. (2008)	?	0	1	0	1	40	1/2	1/2	1	67	50
Sandfeld and Jensen (2005)	?	1	?	0	1	40	1	0	1/2	50	44
Laursen et al. (2000, 2001)*	?	1	?	0	1	40	1/2	0	0	17	31
Waersted et al. (1991)	?	0	1/2	0	1	30	1/2	0	0	17	25

* Data in the two publications concern the same study population and the same experiment and are therefore combined

** Subgroup of total study population that is of interest

*** Experiment 1 included only

^a^(Number of points scored divided by the maximum number of points for the particular category)*100 %

^b^(Number of points scored on all items divided by the maximum number of points)*100 %

^c^This specific item and its scoring is explained in the risk of bias checklist (Table 1)

#### Assessment of heterogeneity

To determine the degree of variation in the true effect sizes and the proportion of the observed variance at each level of analyses, heterogeneity was identified with the *Q* test and *I* ^2^ index, respectively. The degree of heterogeneity was defined as low if *I* ^2^ ≤ 33 %, moderate if 33 % < *I* ^2^ < 67 %, and high if *I* ^2^ ≥ 67 % (Avin and Frey Law 2011), based on previous suggestions (Higgins et al. 2003). Significance level was set at *p* < 0.05.

#### Publication bias

A funnel plot (Duval and Tweedie 2000), consisting of all studies that were included in the meta-analysis, was visually inspected to assess potential publication bias.

## Results

### Search results

The literature search generated a total of 6,484 references: 2,673 in PubMed, 2,698 in EMBASE.com, 995 in PsycINFO, and 118 in The Cochrane Library. After removing duplicates of references that were retrieved from more than one database, 5,390 papers remained. Out of these 5,390 papers, 1,113 met the inclusion criteria at first screening based on title, and 66 based on title and abstract. The full-text of these 66 remaining articles was read to assess for eligibility. From the additional citation search another 22 full-text articles were assessed for eligibility, but no extra studies were included.

**Fig. 2:**
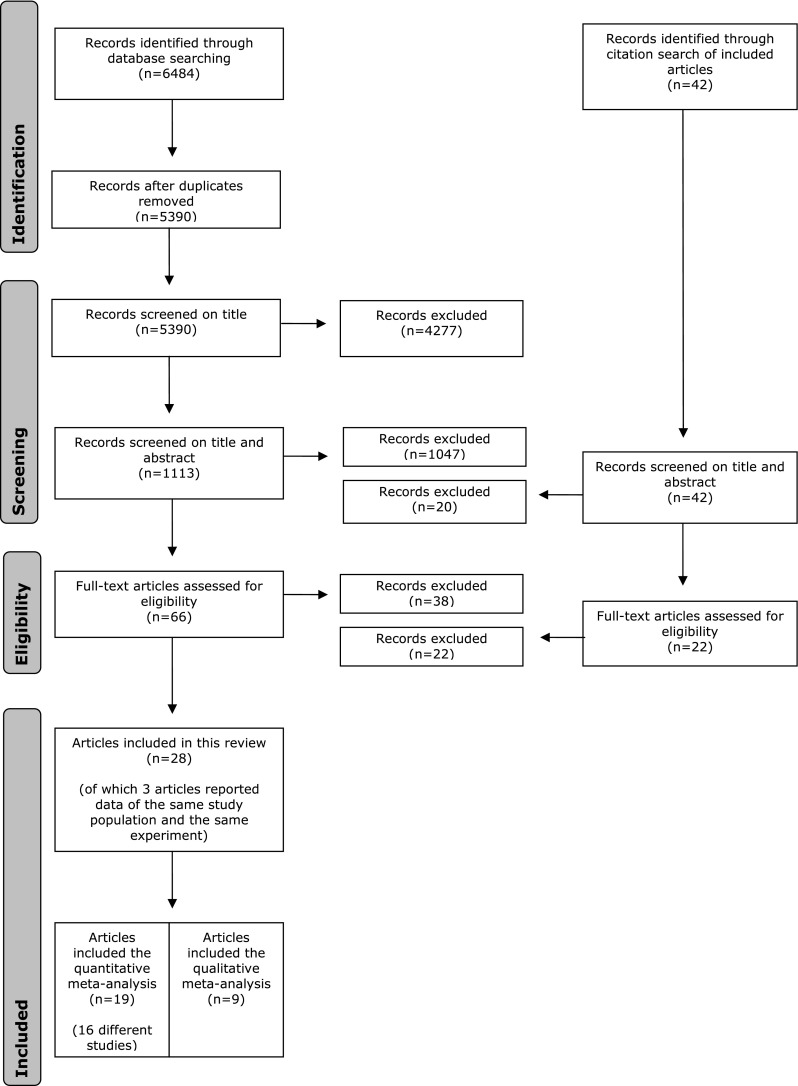
Flow-chart of the search and selection procedure of the studies

Several field studies had to be excluded because muscle activity was only reported on a group-level, including both subjects with and without pain. The final set of 28 included articles consisted of 27 laboratory studies and 1 field study (see Online Resource 2 for the references of all included studies). Of the 28 included articles, 19 articles could be included in the quantitative meta-analysis. A flow chart of the search and selection procedure is presented in Fig. 2.

### Data extraction

From the included articles, we extracted information on study population, study design, computer tasks, stress interventions, and the measured muscles (Online Resource 2). Studies varied in the type of computer tasks that were used, some used more realistic tasks, such as copy typing, keying numbers, data-entry, and realistic mouse work. Others used more constrained computer tasks such as key tapping, key presses, mouse aiming, mouse tracking, or mouse clicking. In addition, there was a large variation in induced stressors. Cognitive/emotional stressors included the color word test, memory task, mental arithmetic task, (two choice) reaction time task, lack of support, surveillance of worker, performance feedback, decrease error rate, simulated office noise, environmental distraction, verbal provocation, and realistic time pressure. Work pace stressors included an increased (or maximal) typing speed, key tapping rate, mouse clicking rate, or a task with a time constraint. Precision demands were induced by reducing target size, increasing mouse gain, or by demanding a high level of clicking accuracy. The majority of the studies measured the trapezius muscle as the neck-shoulder muscle, but some studies included the cervical extensor spinae as well. Studies investigating the effect of stressors on the forearm muscles included one or more of the following muscles: the extensor digitorum, extensor carpi radialis, extensor carpi ulnaris, extensor digitorum communis, flexor carpi radialis, and flexor digitorum superficialis. See the data extraction table for more information (Online Resource 2).

### Risk of bias assessment

The results of the risk of bias assessment are provided in Table 2. Some criteria did not appear to distinguish between studies, i.e. item 1, whether the level of muscle activity before the control and experimental conditions were comparable (i.e. an equal baseline) and item 4, whether an extra motor component was unintentionally introduced by the experimental manipulation. Regarding item 1, none of the articles reported whether there were differences in baseline EMG measurements (even though it was measured in one study). This could have biased the results in these studies. Regarding item 4, none of the studies unintentionally introduced an extra motor component in the experimental manipulation. This issue has not influenced the results.

Thirteen studies had a total score below 60 % of the maximum score and in 12 studies, the total score was above 60 %, indicating a low risk of bias that could have affected the results in these studies.

### Meta-analysis

Nine articles could not be included in the meta-analysis, for several reasons. Data from the field study could not be used in the meta-analysis because the level of stress was not a dichotomous variable but expressed on a scale ranging from 0 to 100 (Blangsted et al. 2003). Four laboratory studies were excluded from the meta-analysis because insufficient information on standard deviations, standard errors, 95 % confidence intervals or *p* values was reported for pooling. Four other studies were excluded for other reasons: (a) only static EMG values (i.e. 5th percentile) were reported (Schnoz et al. 2000), (b) data could not be read accurately from the figure presented (Johnston et al. 2008), and (c) the experimental manipulation was not valid for our comparison (Kristiansen et al. 2009; Sandfeld and Jensen 2005). As to the latter reason, one of the experimental manipulations concerned increased mouse gain (across different target sizes) to enhance movement precision, but this resulted in reduced mouse movement at the same time. The other experimental manipulation (i.e. simulated office noise) had the opposite of the intended effect, during office noise participants reported a lower level of perceived stress and were more in control. In addition, their level of arousal seemed lower compared to the control condition, based on measurements of heart rate, blood pressure, and cortisol. In the data extraction table (Online Resource 2), inclusion in the meta-analysis or reason for exclusion is reported.

Finally, laboratory data of 16 studies, reported in 19 different articles, were included in the meta-analysis (see Online Resource 2, all bold references are included in the meta-analysis). In three cases, two articles were combined because the reported EMG data concerned the same study population and the same experiment.

### Effects of workplace stressors on muscle activity

#### Overall effect of the combined stressor categories on neck–shoulder and forearm muscle activity (Level 1 analysis)

**Fig. 3:**
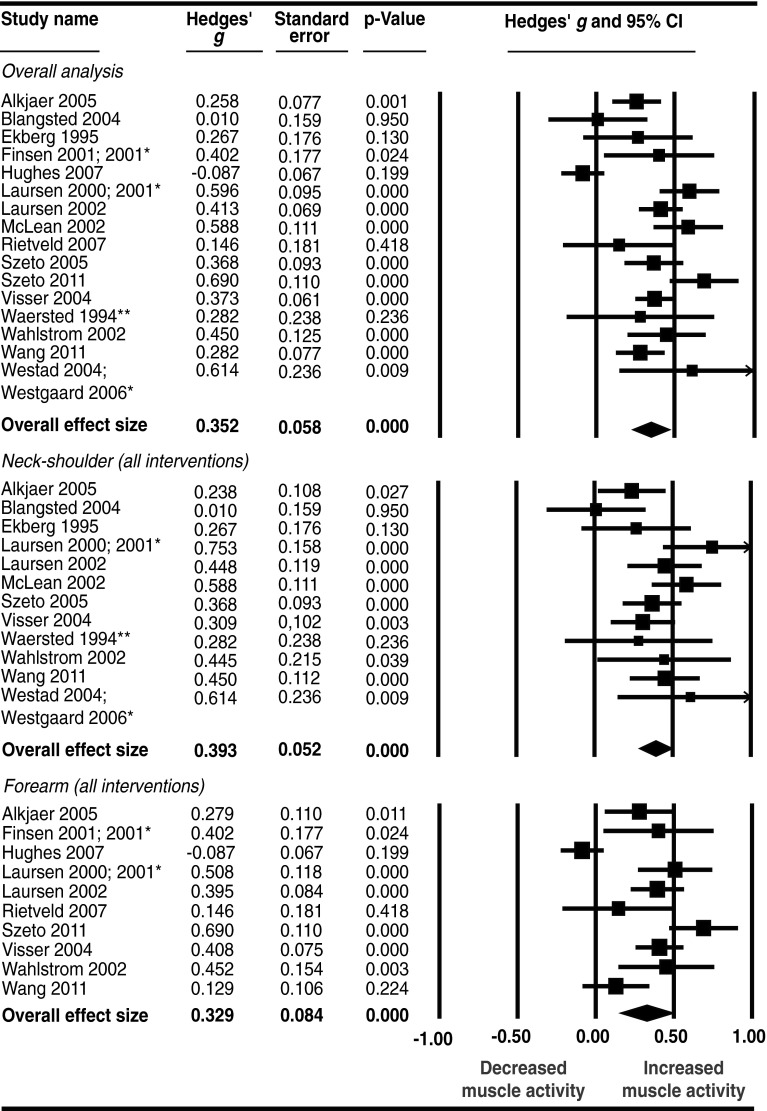
Overall effect of stress on muscle activity of the neck-shoulder and forearm muscles, and the effect on the neck-shoulder and forearm muscles separately:* asterisk* denotes data in the two publications concern the same study population and the same experiment and are therefore combined; *double asterisk* denotes that experiment 1, of four in total, only is included

In the overall meta-analysis, all stressor categories were combined. Ninety five (95) individual effect sizes were synthesized, yielding a statistically significant, medium increase in muscle activity of the neck–shoulder and forearm muscles; Hedges’ *g* = 0.35 (*p* < 0.01) (Fig. 3). The level of heterogeneity was high and significant (*I* ^2^ = 80 %, *Q* = 73.46, *p* < 0.01), indicating that moderating variables are present within this group of experimental manipulations and implying that additional subgroup analyses would be relevant.

All laboratory studies that could not be included in the meta-analyses found increased muscle activity as a result of induced stress, while in the only field study, this effect was not confirmed.

#### Subgroup effect of the combined stressor categories stratified for neck–shoulder and forearm muscle activity (Level 2 analysis)

When examining the effect of all stressor categories on muscle activity stratified to body region (i.e. neck–shoulder muscles and forearm muscles), the effect remained medium; Hedges’ *g* = 0.39 (*p* < 0.01) and 0.33 (*p* < 0.01) for the neck–shoulder and forearm muscles, respectively (Fig. 3). Heterogeneity decreased to moderate in the neck–shoulder subgroup (*I* ^2^ = 42 %), and was not significant according to the *Q* test (*Q* = 19.05, *p* = 0.06). In the forearm subgroup, heterogeneity remained high (*I* ^2^ = 84 %, *Q* = 56.27, *p* < 0.01).

One study that could not be included in the meta-analysis examined the effect of precision on both neck–shoulder and forearm muscle activity, and found a positive effect on forearm muscle activity only.

#### Subgroup effect of stratified stressor categories on neck–shoulder muscle activity (Level 3a analyses)

**Fig. 4:**
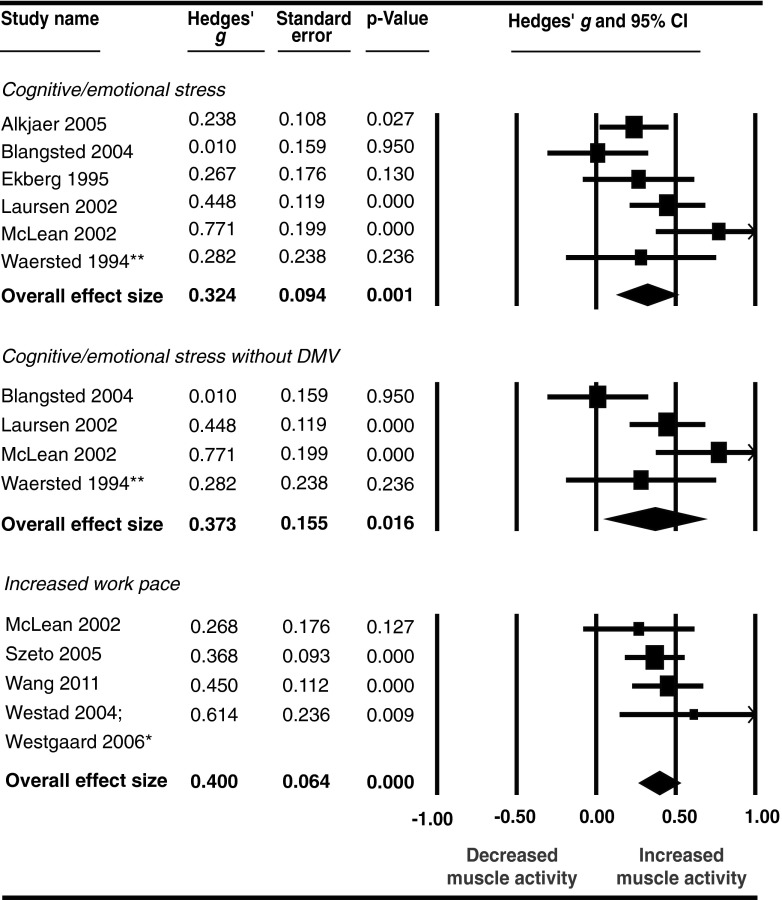
Effect of cognitive/emotional stress and increased work pace on neck-shoulder muscle activity:* asterisk* denotes data in the two publications concern the same study population and the same experiment and are therefore combined; *double asterisk* denotes that experiment 1, of four in total, only is included

The data extraction table (Online Resource 2) reports the stressor categories that each study was assigned to. The effect of stress on neck–shoulder muscle activity was somewhat smaller for non-task interfering cognitive and emotional stressors than for task interfering work pace stressors; Hedges’ *g* of 0.32 (*p* < 0.01) and 0.40, (*p* < 0.01), respectively (Fig. 4). A difference in level of heterogeneity was found for both subgroups which was moderate but not significant in the cognitive/emotional stress subgroup (*I* ^2^ = 54 %, *Q* = 10.77, *p* = 0.06), and absent in the subgroup with work pace stressors (*I* ^2^ = 0 %, *Q* = 1.70, *p* = 0.64). Performing a subgroup analysis of the neck–shoulder subgroup involving precision as a stressor was not possible because only two studies were included in this group.

When including only those studies in which movement velocity was not decreased during the cognitive and emotional stress interventions, the Hedges’ *g* slightly increased from 0.32 (*p* = 0.03) to 0.37 (*p* = 0.02) (Fig. 4). Heterogeneity was high, *I* ^2^ = 69 % (*Q* = 9.74, *p* = 0.02).

Four studies that could not be included in the meta-analysis examined the effect of cognitive/emotional stress on neck–shoulder muscle activity. All four studies found increased muscle activity as a result of the stressor. The one field study that could not be included in the meta-analysis did not find a statistically significant effect on neck–shoulder muscle activity in workers with high workplace stress.

#### Subgroup effect of stratified stressor categories on forearm muscle activity (Level 3b analyses)

**Fig. 5:**
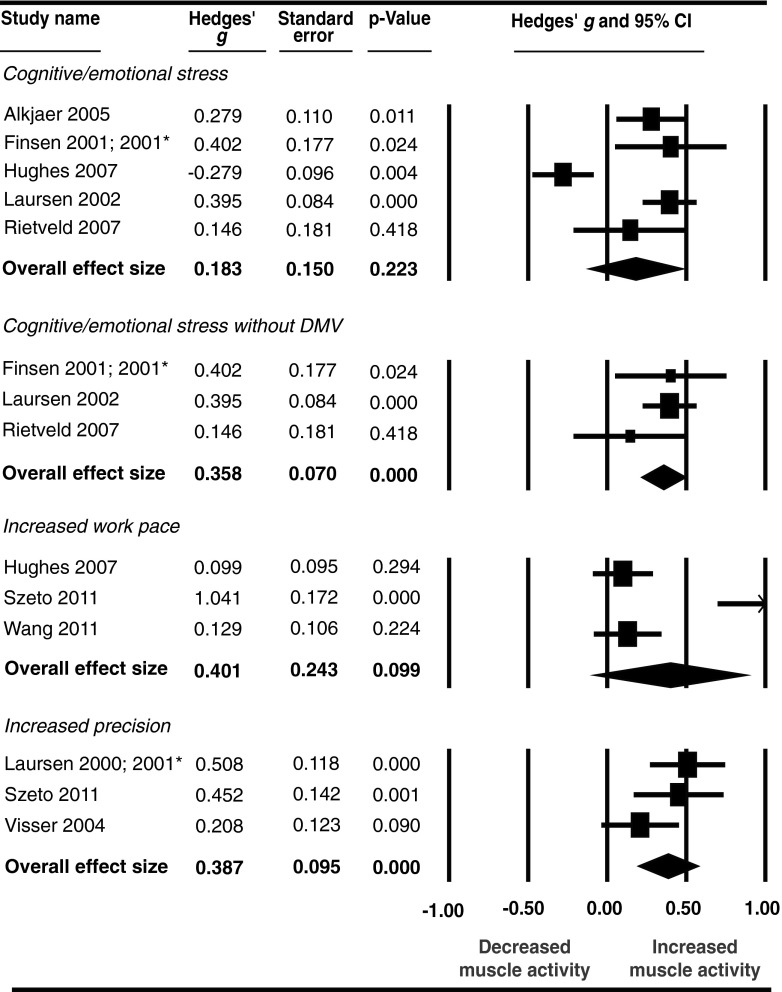
Effect of cognitive/emotional stress, increased work pace, and increased precision on forearm muscle activity:* asterisk* denotes data in the two publications concern the same study population and the same experiment and are therefore combined

A small, but not significant, effect of cognitive and emotional stressors was found on forearm muscle activity, Hedges’ *g* = 0.18 (*p* = 0.22), whereas for work pace and precision stressors medium effects were found with Hedges’ *g* of 0.40 (*p* = 0.09) and 0.39 (*p* < 0.01), respectively (Fig. 5). Except for the subgroup with precision as stressor, heterogeneity was high (cognitive/emotional stress *I* ^2^ = 87 %, *Q* = 31.57, *p* < 0.01; work pace *I* ^2^ = 92 %, *Q* = 24.91, *p* < 0.01; precision *I* ^2^ = 41 %, *Q* = 3.38, *p* = 0.19).

When selecting only those studies in which movement velocity did not decrease as a result of the cognitive and emotional stress intervention, the Hedges’ *g* changed from 0.18 (*p* = 0.22) in the overall analyses to 0.36 (*p* < 0.01) in the analyses in which only studies were selected in which this adaptation strategy did not occur. In addition, heterogeneity decreased and was not present in this latter subgroup analysis: *I* ^2^ changed from 87 to 0 % (*Q* = 1.62, *p* = 0.44).

**Fig. 6:**
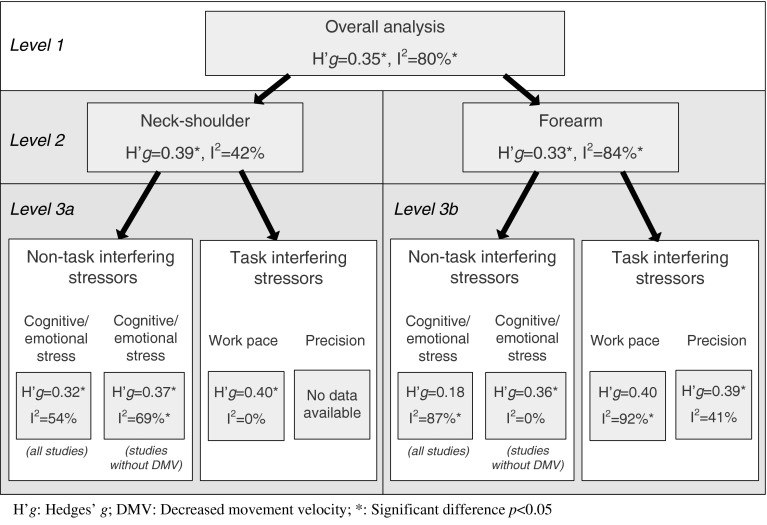
Summary of effect sizes and heterogeneity per subgroup

Two studies that could not be included in the meta-analysis examined the effect of increased work pace on forearm muscle activity. Both studies reported increased muscle activity. For an overview of all the results of the meta-analyses, see Fig. 6.

### Sensitivity analysis

To assess whether the results of the meta-analysis were affected by bias, we compared the results of studies with a relatively low risk of bias (i.e. a total score ≥60 % in the risk of bias assessment) to the results of studies with a higher risk of bias (total score <60 %). This sensitivity analysis revealed a smaller effect size for the studies with a relatively low risk of bias (9 studies) than for the studies with a higher risk of bias (7 studies), Hedges’ *g* of 0.24 (*p* = 0.01) and 0.53 (*p* < 0.01), respectively.

### Publication bias

From the visual inspection of a funnel plot, it was concluded that the results were unlikely to be adversely affected by publication bias, indicated by a symmetrical distribution of the studies and the unchanged effect size when adjusted for missing studies.

## Discussion

### Summary of main results

The aim of this review was to summarize the evidence supporting the relationship of workplace stressors and increased muscle activity in the neck–shoulder and forearm region during (simulated) computer work. The meta-analysis, combining the effects of all types of stress interventions, yielded a medium significant effect on muscle activity of the neck–shoulder and forearm muscles. In line with this finding, all 8 laboratory studies that were not included in the meta-analyses found an increased muscle activity as a result of induced stress. However, the only field study, which was not included in the meta-analyses, did not confirm this effect.

In answer to our second research question, neck–shoulder and forearm muscle activity were not differently affected by non-task interfering stressors, such as cognitive and/or emotional demands, and task interfering stressors, such as an increased work pace and/or precision.

### Interpretation of results

We expected that the neck–shoulder muscles would be less affected by task interfering stressors than the forearm muscles, because the main function of these muscles is to control posture and they are not directly involved in the operational movements during computer work. However, it can be argued that having people work fast or precisely would also have effects that are not directly task related. It is likely that instructing people to increase their work pace or level of precision would simultaneously increase their level of arousal, which was also suggested by Visser et al. (2004). Two of the four studies included in the work pace subgroup reported the level of arousal (measured by heart rate and blood pressure), and indeed found it to be increased in the experimental condition (Wang et al. 2011; Westad et al. 2004; Westgaard et al. 2006). Possibly both stress types induce a similar increase in arousal. This could explain the medium increase in neck–shoulder muscle activity as a result of task interfering stress that we found, which was comparable to the effect of the non-task interfering stress.

Furthermore, we hypothesized that forearm muscle activity would be mainly increased by task interfering stressors, because the forearm muscles are controlling the operating hands during the performance of a computer task. As expected, task interfering stress led to a medium increase in forearm muscle activity. Unexpectedly, non-task interfering stressors also revealed a medium increase in forearm muscle activity. This could be explained by the neuromotor noise theory (van Gemmert and van Galen 1997). It has been suggested that cognitive and emotional stressors increase neuromotor noise, resulting in a larger kinematic variability during task performance. In order to meet task demands such as high precision, this kinematic variability needs to be suppressed. This can be done by increasing limb stiffness through co-contraction of the agonist and antagonist muscles (Selen et al. 2005). In an experimental study, Van Loon et al. (2001) indeed show that limb stiffness was higher with higher mental stress. In this way, higher forearm muscle activity may not only be the result of task interfering stressors but also indirectly be the result of higher cognitive and emotional stressors.

The level of statistical heterogeneity of our main result was high according to the I^2^ index (*I* ^2^ = 80 %), indicating that the effects on muscle activity differed across studies. In our main analysis, all different stressor types, computer tasks, and muscles (e.g. neck–shoulder muscles, forearm extensors, and flexors) from the individual studies were grouped together. It is likely that there are still underlying variables present within this group that are responsible for increasing or reducing the effect on muscle activity. We tried to eliminate the effects of such potential underlying, moderating variables by performing additional subgroup analyses. This was successful in some cases. For example, the subgroup analysis of work pace stressors on neck–shoulder muscle activity resulted in a statistically homogeneous group. All four studies that we included in this group examined a highly comparable stress intervention and computer task (i.e. an increased typing speed during copy typing). In addition, selecting only studies in which movement velocity was not decreased as an adaptation strategy for cognitive and emotional stressors resulted in a large drop in heterogeneity (from 87 to 0 %) for forearm muscle activity.

### Bias of included studies

Overall, the risk of bias of the included studies was rather low, since more than half of the studies scored more than 60 % in the risk of bias assessment. When comparing the results of studies with a high and lower quality of evidence (i.e. low and higher risk of bias), we saw a decrease in our overall effect for the studies with a high quality of evidence (Hedges’ *g* 0.35–0.24) and an increase of the overall effect for the studies with a lower quality of evidence (Hedges’ *g* 0.35–0.53). The two groups mainly differed on one item, i.e. the computer task performed. The group of studies with a lower risk of bias, examined more realistic and complex computer tasks (e.g. copy typing with ten fingers, data-entry on numerical part of the keyboard with dominant hand, and realistic mouse tasks). In the group of studies with a higher risk of bias more simple and constrained computer tasks were used (e.g. key presses with one finger, data-entry with one finger, and structured mouse tasks at predefined speed). As a result, in the first group participants had more freedom in task performance and thereby more possibilities to adapt their task performance to cope with the stressor. Given the above results, the results reported in this review may be an overestimation of the effect of workplace stressors on muscle activity in real life.

### Strengths and limitations

Several strengths of the present review are noteworthy. To our knowledge, this is the first review to summarize the evidence of the effect of workplace stressors, mostly simulated in a laboratory setting, on neck–shoulder and forearm muscle activity. Furthermore, to avoid sources of bias in the review process, two reviewers independently selected studies for inclusion, performed the risk of bias assessment, and extracted the data that were used in the meta-analyses. Especially, data extraction was not always straightforward, because different choices to define the reference and experimental conditions could be made. Sometimes multiple stress levels or multiple interventions were tested in one study. Different reference and experimental conditions were defined by the two reviewers in only 8 of the 95 cases. Agreement was reached in a consensus meeting. Overall, agreement on selection, risk of bias assessment, and data extraction were high and therefore, we do not expect that this biased our results much. Moreover, the criteria for including studies in this review were strict. In this way, the studies that were grouped together in the meta-analyses can be considered as homogeneous in terms of experimental design. Therefore, despite a high level of statistical heterogeneity in some analyses, we are of the opinion that the observed effect can still be interpreted as relevant.

A first limitation in this review concerns our inability to include all studies in the meta-analyses because insufficient information on muscle activity data was reported. In addition, in four articles muscle activity was needed to be read from figures, which was somewhat inaccurate. However, as mentioned above, data extraction was done independently by both reviewers and differed little between the two. Furthermore, data of field studies are lacking in this review. Only one field study could be included, which may negatively have influenced the generalizability of our results to the actual workplace. A last limitation is that this review mainly reports effects of individual stressors, whereas in a realistic work setting most often a combination of stressors will be present.

### Implications of results

Unfortunately, the clinical relevance of the increase in muscle activity remains unclear, since the effect size (Hedges’ *g*) cannot be transformed into physiologically interpretable units (such as  % MVC). However, following the Cinderella hypothesis (Hagg 1991), even small increases in muscle activity could lead to a serious burden for small muscle fibers that are continuously active. The overload of muscle fibers can cause muscle damage and eventually chronic pain (Visser and van Dieen 2006).

Based on the current findings, it is difficult to predict the effect of workplace stressors in a realistic work setting, since only one field study was included and laboratory stressors do not correspond directly to real field stressors. In the sensitivity analysis, we found that for laboratory studies with more realistic work tasks the effect size was lower than for laboratory studies using more constrained work tasks. Realistic computer tasks likely impose less constraints on task performance allowing more freedom to cope with stressors, which might explain the smaller increase of muscle activity. However, it can be questioned whether such degrees of freedom would be used in the real work setting. For example, when a cognitively demanding task needs to be performed under high time pressure, decreasing work pace, a commonly used adaptation strategy in the reviewed laboratory studies, might not be an acceptable adaptation strategy, and muscle activity may be even higher than that found in this review. Based on this reasoning, we suggest that work pace may be a more important stressor in terms of its effect on muscle activity than cognitive/emotional stressors. However, field studies are needed to reveal the effect of stressors and coping strategies used in real life.

Moreover, large inter-individual differences in muscle activity as a response to stressors have been found (Mclean and Urquhart 2002; Waersted et al. 1991; Westgard et al. 1993). It has been indicated that high motivation increases the effect of workplace stressors on muscle activity (Waersted et al. 1994; Waersted and Westgaard 1996), making it is plausible that the increase in muscle activity will be larger for people with high motivation for their job compared to people with low motivation. This would mean that our findings might be an underestimation for highly motivated individuals. However, hardly any of the included studies studied motivational aspects or personality traits, and therefore, determining the moderating effect of motivation to perform well or personality on the effect of workplace stressors on muscle activity is currently impossible. Therefore, it is recommended that future field studies include the aspect of motivation and participants with different personality traits.

## Conclusion

Simulated workplace stressors resulted in a medium increase in neck–shoulder and forearm muscle activity. No indications were found that different types of stressors affect muscle activity in these body regions differently. These conclusions are fully based on laboratory studies, since field studies investigating the effects of realistic workplace stressors in a real-life work setting are currently lacking.

## Electronic supplementary material

Below is the link to the electronic supplementary material.

Supplementary material 1 (PDF 20 kb)

Supplementary material 2 (PDF 69 kb)
