# Supplementary material for: The effects of workplace stressors on muscle activity in the neck-shoulder and forearm muscles during computer work: a systematic review and meta-analysis
Source: Eur J Appl Physiol. 2013 Mar 5;113(12):2897–912. doi: 10.1007/s00421-013-2602-2 (PMC3828497; doi:10.1007/s00421-013-2602-2)
Supplement: Supplementary file 1 — Supplementary material 1 (PDF 20 kb) [file 421_2013_2602_MOESM1_ESM.pdf]

The effects of workplace stressors on muscle activity in the neck-shoulder and forearm muscles during computer work: a systematic review and meta-analysis. Eur J App Physio. B.H.W. Eijckelhof, M.A. Huysmans, J.L. Bruno Garza, B.M. Blatter, J.H. van Dieën, J.T. Dennerlein, A.J. van der Beek.

Corresponding Author: Dr. M.A. Huysmans, E mail: m.huysmans@vumc.nl, a) Department of Public and Occupational Health and the EMGO Institute for Health and Care Research, VU University Medical Center, Van der Boechorststraat 7, 1081 BT, Amsterdam, The Netherlands, b) Body@Work Research Center on Physical Activity, Work and Health, TNO-VU/VUmc, Amsterdam, The Netherlands.

Online Resource 1 Search strategy in PubMed

Search	PubMed 04-04-2011	Result
#10	Search #9 NOT (animals[mh] NOT humans[mh])	**2673**
#9	Search #8 NOT ("addresses"[Publication Type] OR "biography"[Publication Type] OR "comment"[Publication Type] OR "directory"[Publication Type] OR "editorial"[Publication Type] OR "festschrift"[Publication Type] OR "interview"[Publication Type] OR "lectures"[Publication Type] OR "legal cases"[Publication Type] OR "legislation"[Publication Type] OR "letter"[Publication Type] OR "news"[Publication Type] OR "newspaper article"[Publication Type] OR "patient education handout"[Publication Type] OR "popular works"[Publication Type] OR "congresses"[Publication Type] OR "consensus development conference"[Publication Type] OR "consensus development conference, nih"[Publication Type] OR "practice guideline"[Publication Type])	3060
#8	Search #6 OR #7	3065
#7	Search #1 AND #4 AND #5	840
#6	Search #1 AND #2 AND #3 AND #5	2673
#5	Search "Mental Competency"[Mesh] OR "Mental Health"[Mesh] OR "Mental Processes"[Mesh] OR "Personal Autonomy"[Mesh] OR "Psychology, Applied"[Mesh] OR "Psychophysiology"[Mesh] OR "Job Satisfaction"[Mesh] OR "job satisfaction"[tiab] OR "time pressure"[tiab] OR "verbal provocation"[tiab] OR "verbal provocations"[tiab] OR precision[tiab] OR cognitive[tiab] OR cognition[tiab] OR stroop[tiab] OR mental[tiab] OR multitask*[tiab] OR "memory demand"[tiab] OR "decision demand"[tiab] OR "memory demands"[tiab] OR "decision demands"[tiab] OR pace[tiab] OR pacing[tiab] OR stressor*[tiab] OR "Social Environment"[Mesh] OR "social environment"[tiab] OR "social environments"[tiab] OR "psychosocial environment"[tiab] OR "psychosocial environments"[tiab] OR "reaction time"[tiab] OR "reaction-time test"[tiab] OR "psychosocial factor"[tiab] OR "psychosocial factors"[tiab] OR "psychological factor"[tiab] OR "psychological factors"[tiab] OR "psychological stress"[tiab] OR "psychological stresses"[tiab]	1452775
#4	Search "Neck Muscles"[Mesh] OR trapezius[tiab] OR trapezii[tiab] OR "extensor carpi"[tiab] OR "Head Movements"[Mesh] OR "head movement"[tiab] OR "head movements"[tiab] OR "head tilt"[tiab] OR "head tilting"[tiab] OR "head flexion"[tiab] OR "head extension"[tiab] OR "neck flexion"[tiab] OR "neck extension"[tiab] OR "shoulder rotation"[tiab] OR abduction[tiab] OR adduction[tiab] OR "arm movement"[tiab] OR "arm movements"[tiab] OR ulnar[tiab] OR radial[tiab]	77281
#3	Search "Muscle Strength"[Mesh] OR muscle[tiab] OR muscles[tiab] OR muscular[tiab] OR electromyograph*[tiab] OR EMG[tiab] OR "forward bending"[tiab] OR "side bending"[tiab] OR "Muscle Tonus"[Mesh] OR "Muscle Fatique"[Mesh] OR "Muscle Contraction"[Mesh] OR "Recruitment,	2166179

Neurophysiological"[Mesh] OR "Physical Exertion"[Mesh] OR "Adaptation, Physiological"[Mesh:NoExp] OR "Exercise Tolerance"[Mesh] OR "Biomechanics"[Mesh] OR biomechanic*[tiab] OR "Kinetics"[Mesh] OR "Mechanical Processes"[Mesh:NoExp] OR "Task Performance and Analysis"[Mesh] OR "Pressure"[Mesh:NoExp] OR "Musculoskeletal System"[Mesh] OR musculoskeletal[tiab] OR "Posture"[Mesh:NoExp] OR posture[tiab] OR postures[tiab] OR postural[tiab] OR "Postural Balance"[Mesh] OR "Range of Motion, Articular"[Mesh] OR "range of motion"[tiab] OR "Movement"[Mesh:NoExp] OR "Motor Activity"[Mesh:NoExp] OR "Myography"[Mesh] OR myograph*[tiab] OR "Work Capacity Evaluation"[Mesh] OR "Stress, Mechanical"[Mesh] OR "mechanical stress"[tiab] OR "mechanical stresses"[tiab] OR "Workload"[Mesh] OR flexion[tiab] OR extension[tiab]

#2 Search "Upper Extremity"[Mesh] OR "Upper Extremity"[tiab] OR "Upper Extremities"[tiab] OR "Upper Limb"[tiab] OR "Upper Limbs"[tiab] OR shoulder[tiab] OR shoulders[tiab] OR arm[tiab] OR arms[tiab] OR elbow[tiab] OR elbows[tiab] OR forearm*[tiab] OR wrist[tiab] OR wrists[tiab] OR hand[tiab] OR hands[tiab] OR finger[tiab] OR fingers[tiab] OR "Neck"[Mesh] OR neck[tiab] OR necks[tiab] OR head[tiab] OR heads[tiab] 754420

#1 Search "Office Automation"[Mesh] OR office OR "Computers"[Mesh] OR computer*[tiab] OR "desk work"[tiab] OR VDU[tiab] OR VDT[tiab] OR pc[tiab] OR keyboard[tiab] OR mouse[tiab] OR mousing[tiab] OR typing[tiab] OR keying[tiab] OR "professional occupation"[tiab] OR "professional occupations"[tiab] OR sitting[tiab] OR "seated position"[tiab] OR "workplace"[Mesh] OR workplace[tiab] OR workplaces[tiab] OR "Employment"[Mesh] OR employment[tiab] OR employments[tiab] OR "repetitive work"[tiab] OR "assembly work"[tiab] OR "assembly worker"[tiab] OR "assembly workers"[tiab] OR cashier[tiab] OR cashiers[tiab] OR "supermarket employee"[tiab] OR "supermarket employees"[tiab] OR "white collar work"[tiab] OR "white collar worker"[tiab] OR "white collar workers"[tiab] OR "professional worker"[tiab] OR "professional workers"[tiab] OR "static work task"[tiab] OR "static work tasks"[tiab] OR "low force work"[tiab] OR "low force task"[tiab] OR "low force tasks"[tiab] 828621
