# Supplementary material for: The effects of workplace stressors on muscle activity in the neck-shoulder and forearm muscles during computer work: a systematic review and meta-analysis
Source: Eur J Appl Physiol. 2013 Mar 5;113(12):2897–912. doi: 10.1007/s00421-013-2602-2 (PMC3828497; doi:10.1007/s00421-013-2602-2)
Supplement: Supplementary file 2 — Supplementary material 2 (PDF 69 kb) [file 421_2013_2602_MOESM2_ESM.pdf]

The effects of workplace stressors on muscle activity in the neck-shoulder and forearm muscles during computer work: a systematic review and meta-analysis. Eur J App Physio. B.H.W. Eijckelhof, M.A. Huysmans, J.L. Bruno Garza, B.M. Blatter, J.H. van Dieën, J.T. Dennerlein, A.J. van der Beek.
Corresponding Author: Dr. M.A. Huysmans, E mail: m.huysmans@vumc.nl, a) Department of Public and Occupational Health and the EMGO Institute for Health and Care Research, VU University Medical Center, Van der Boechorststraat 7, 1081 BT, Amsterdam, The Netherlands, b) Body@Work Research Center on Physical Activity, Work and Health, TNO-VU/VUmc, Amsterdam, The Netherlands.

Online Resource 2 Data extraction table with characteristics of studies (*n* = 28) included in this review

Study	Study population		Study design		Control condition	Intervention	Wash-out period		Measured muscles	Meta-analysis			
	Number of participants	Characteristics			Task	Added stressor	Time between control and intervention session	Duration of sessions	Forearm, neck-shoulder	Inclusion	Subgroup category	Adaptation strategy to induced stressor(s), e.g. DMV	Inclusion subgroup analysis cognitive / emotional stressors without DMV
**Alkjaer et al. 2005**	28F	experienced computer workers	lab	exp CO	mouse clicks at predefined pace	CWT, answer as quickly and correctly as possible (same predefined cue rate as in CON), performance feedback	30 min	30 min	ED, FCR TRAP, NECK	Y	cognitive load	DMV: Y clicking frequency<br><br>number of correct answers: Y<br><br>response time: Y	N
Blangsted et al. 2003**	24F		field	obs	participants' own computer work	Realistic time pressure and work load	NA	NA	EDC TRAP	N stress level not dichotomous	NA	NA	NA
**Blangsted et al. 2004**	12F	experienced computer workers	lab	exp CO	keying numbers with dominant hand on numerical part of the keyboard	MT, lack of support, surveillance of worker	8 min	12 min	TRAP	Y	cognitive load, emotional stress	DMV: N<br><br>number of errors: N	Y

Bloemsaat et al. 2005	9F, 5M	mean age 26, experienced computer workers	lab	exp CO	perform rapid tapping movements with dominant index finger	1.MT<br>2.increased tapping rate	1. 1 min<br>2. no pause	1. ±9 min<br>2. ±4,5 min	ED, ECR, ECU, FDS TRAP	N<br>missing data	NA	1: DMV: N predefined<br><br>2: DMV: N (= intervention)	NA
**Ekberg et al. 1995 (pilot)**	10M	mean age 25 (20-34), students	lab	exp CO	data entry task on numerical part of the keyboard with dominant hand; self-selected pace, as correct as possible	1.MA, as quick and correct as possible<br><br>2.CWT, at predetermined pace as correct as possible	40 sec	80 sec	TRAP	1. Y<br><br>2. N<br>task differed from control condition	1.cognitive load<br><br>2.cognitive load	1. DMV: Y<br><br>2. DMV: Y	1.N<br><br>2.N
**Ekberg et al. 1995 (main)**	20F	mean age 36 (19-56), professional computer workers	lab	exp CO	data entry task on numerical part of the keyboard with dominant hand; self-selected pace, as correct as possible	MA, as quick and correct as possible	different part of the day (morning-noon)	1 hour	TRAP	Y	cognitive load	DMV: Y	N
**Finsen et al. 2001; Finsen et al. 2001***	9F	mean age 25 (20-35), regular computer users, students	lab	exp CO	mouse task with right hand, typing numbers on numerical part of the keyboard with left index finger, avoid errors	MT	?	CON: 7 min<br>EXP: 1 hour	EDC, ECR, FDS	Y	cognitive load	DMV: N predetermined<br><br>number of errors: Y	Y
Gerard et al. 2002	16F, 2M	mean age 34 (22-57), experienced 10-finger typists	lab	exp CO	copy typing with two hands, self paced	increased typing speed, 100% max	at least a day	30 min	Finger extensor, Finger flexor	N<br>missing data	NA	DMV: N (= intervention)	NA

**Hughes et al. 2007**	9F, 9M	mean age 25,4 (± 6,4), 10-finger touch typists, students	lab	exp CO	copy typing, errors no issue	1.verbal MA<br><br>2.increased typing speed	3 min	5 min	ECU, FCU	1.Y<br><br>2. Y	1.cognitive load<br><br>2.work pace	1. DMV: Y<br><br>2. DMV: N (=intervention)<br><br>typing errors: Y	1.N<br><br>2.NA
Johnston et al. 2008	55F	33 working, 22 non-working, competent typists	lab	exp	copytyping at comfortable pace	type as fast and accurately as possible with superimposed stress (surveillance of worker, performance feedback)	a few minutes (or long enough for heart rate to return to baseline level)	5 min	TRAP, CES	N<br>not possible to extract data accurately from the presented figure	NA	DMV: Y<br><br>error rate: Y	NA
Kristiansen et al. 2009	10F	mean age 34,7 (25-51), experienced computer workers	lab	exp CO	1.mouse work<br><br>2.order judgment task, answer by key press with index finger of the dominant hand	simulated office noise	12 min	20 min (task 1+2 together; 10 min per task)	TRAP	1.N<br>no valid intervention for our comparison<br><br>2.N<br>no valid intervention for our comparison	1.NA<br><br>2.NA	DMV: N<br><br>response time: N<br>number of errors: N	1.NA<br><br>2.NA
**Laursen et al. 2000; Laursen et al. 2001***	17F	mean age young group: 25 (22-28), elderly group: 63 (56-70), experienced computer workers	lab	exp CO	mouse tasks at predefined speed	reduced target size	1 hour<br>(10 min break, 50 min performing mouse work)	100 sec	ECR, FCR, ED TRAP, NECK	Y	precision	DMV: N<br>predefined speed,<br><br>error rate: Y	NA

**Laursen et al. 2002**	12F	mean age 32 (27-41), experienced computer users	lab	exp CO	key presses and mouse clicks, predefined reaction time	CWT, as quickly (within same reaction time as in control condition) and as correctly as possible, performance feedback	2 min	8 min	ECR, ED, ECU, FCU TRAP, NECK	Y	cognitive load	DMV: N<br><br>response time for correct answer: Y	Y
Leyman et al. 2004	4F, 9M	mean age 33 (22-44), touch typists	lab	exp CO	copytyping	MT	3 min	±1 min	TRAP, CES	N missing data	NA	DMV: N<br><br>number of characters typed correctly: Y	NA
**McLean and Urquhart 2002**	6F, 4M	mean age 23 (± 2), experienced computer users	lab	exp CO	1.copytyping at comfortable pace	1.1 maximal typing speed<br><br>1.2.maximal typing speed, environmental distraction, surveillance of worker, performance feedback	30 min	30 min	TRAP	Y	1.1 work pace<br><br>1.2 work pace, emotional stress	1.1. DMV: N (= intervention)<br><br>1.2. DMV: N (= part of intervention), but lower than in first intervention (1.1.)	1.1.NA<br><br>1.2. N no individual stressor as intervention
					2.copytyping at maximal speed	2.environmental distraction, surveillance of worker, performance feedback					2. emotional stress	2. DMV: N	2.Y
**Rietveld et al. 2007**	20	mean age 38 (23-49)	lab	exp CO	copytyping after relaxation break	copytyping after superimposed stress	15 min	9 min	ED	Y	emotional stress	DMV: N	Y
Sandfeld and Jensen 2005	17F, 16M	mean age young group: 26,5 (± 2,5), elderly group: 64,9 (± 3,6), experienced computer users	lab	exp CO	mouse pointing task at constant (predefined) clicking frequency, errors are no issue	increased mouse gain across different target sizes	3 min	3,5 min	ECR, ED, ECU, FCR TRAP, NECK	N no valid intervention for our comparison	NA	DMV: N<br><br>% correct hits: Y	NA

Schnoz et al. 2000	2F, 7M	mean age 29 (± 4)	lab	exp CO	key tapping with index finger of the dominant hand	increased tapping rate	?	8 sec	ED, FD TRAP	N only static EMG values presented	NA	DMV: N (= intervention)	NA
**Szeto et al. 2005****	20F	mean age 30,4 (22-42), office workers	lab	Exp CO	copy typing at normal typing speed, correct typing errors (>10% errors not corrected, program would stop)	Increased typing speed (at least 20%)	?	20 min	TRAP, CES	Y	work pace	DMV: N (= intervention)	NA
**Szeto and Lin 2011****	8F	mean age 26,5 (23-30), experienced typists, office workers	lab	exp CO	mouse tasks 1.comfortable clicking speed, precision was set at medium level	1.maximal clicking speed, precision was set at medium level	3 min	5 min	ECU, ECR, FCU, FCR	Y	1.work pace	1. DMV: N (= intervention)	1.NA
					2.low level of clicking accuracy, self-selected work pace	2.high level of clicking accuracy, self-selected work pace					2.precision	2. DMV: Y	2.NA

**Visser et al. 2004**	6F, 4M	age 23-58, experienced computer workers	lab	exp CO	mouse tasks: tracking and aiming.<br>low level of clicking accuracy, low mental pressure	1.high level of clicking accuracy	?	2 min	ED, FD TRAP	1.Y	1. precision	1.aiming: DMV: Y error rate: Y<br><br>tracking: DMV: ?<br>error rate: Y	1.aiming: NA<br><br>tracking: NA
						2.high mental pressure; perform as:<br>fast (*in aiming task*)<br>correct (in *tracking task*)<br>as possible, performance feedback				2.Y	2. emotional, work pace, precision	2.aiming: DMV: N<br>(= part of intervention)<br>error rate N<br><br>tracking: DMV: ? error rate: N	2.aiming: N emotional stress not an individual stressor<br><br>tracking: N emotional stress not an individual stressor
						3.high level of clicking accuracy, high mental pressure				3.Y	3. precision, emotional, work pace	3.aiming: DMV: N<br>(= intervention)<br>error rate N<br><br>tracking: DMV: ? error rate: Y	3.aiming: N emotional stress not an individual stressor<br><br>tracking: N emotional stress not an individual stressor
Waersted et al. 1991	17F, 1M	median age 24 (21-31)	lab	exp CO	simple RT, varying response-stimulus interval 1,5-3,5 sec, key presses for answering, respond as fast as possible	complex RT, constant response-stimulus interval 1,5 sec, respond as fast and correctly as possible	±30 sec (to start the next task)	±5 min	TRAP	N missing data	NA	DMV: Y	NA

**Waersted et al. 1994*** **	13F	median age 22 (19-30), students	lab	exp CO	key presses with right index finger in complex two-choice reaction time task, instructed to work fast and make few mistakes	feedback on speed	90 sec	6 min	TRAP	Y	Cognitive load	DMV: N (= part of intervention)<br><br>error rate: Y	Y
**Wahlström et al. 2002**	7F, 8M	mean age 30 (18-48), experienced computer mouse users	lab	exp CO	mouse and keyboard work (text editing), 2 pages, no time constraint	work as fast as possible, 4 pages, time constraint, verbal provocation	?	?	ED TRAP	Y	emotional stress, work pace	DMV: N (= intervention)	N emotional stress not an individual stressor
**Wang et al. 2011**	7F, 7M	mean age 23,2 (± 3), experienced typists, university students	lab	exp CO	copytyping at low speed: 60 Hz	copytyping with increased speed: 180 Hz	no pause	5 min	ECR, FCU TRAP, CES	Y	work pace	DMV: N (= intervention)	NA
**Westad et al. 2004; Westgaard et al. 2006***	11F, 9M	age 20-56	lab	exp CO	copy typing	copytyping with 10% increased speed while maintaining or improving error rate	2 min	10 min	TRAP	Y	work pace	DMV: ?	NA

*: data in the two publications concern the same study population and the same experiment and are therefore combined; ** subgroup of total study population that is of interest; ***: experiment 1 included only; F: female; M: male; lab: laboratory; exp: experimental; obs: observational; CO: cross-over design; MT: memory task; MA: mental arithmetic task; CWT: color word test; RT: (two choice) reaction time task; min: minutes; ED: extensor digitorum; ECR: extensor carpi radialis; ECU: extensor carpi ulnaris; EDC: extensor digitorum communis; FCR: flexor carpi radialis; FDS: flexor digitorum superficialis; TRAP: trapezius; NECK: neck extensor muscle group; CES: cervical extensor spinae; DMV: decreased movement velocity; Y: yes; N: no; ?: don't know; NA: not applicable
